# GeoRescue: A Geometric LiDAR Point Cloud Registration Framework for Resource-Constrained Edge Platforms

**DOI:** 10.3390/s26113422

**Published:** 2026-05-28

**Authors:** Yuyu Sun, Zongkai Shang, Mingxiao Yang, Fandi Meng, Mengxuan Mu, Heqi Yan

**Affiliations:** College of Computer Science and Technology, Changchun University, Changchun 130022, China; 13386378939@163.com (Z.S.); 19806085316@163.com (M.Y.); 19842806202@163.com (F.M.); mumengxuan2025@163.com (M.M.); 231502546@mails.ccu.edu.cn (H.Y.)

**Keywords:** LiDAR point cloud registration, robotics perception, training-free geometric registration, edge sensing, low-overlap registration, resource-constrained platforms

## Abstract

**Highlights:**

**What are the main findings?**

- A training-free geometric method is proposed for LiDAR point cloud registration on resource-constrained edge platforms.
- The modular pipeline synergistically combines asymmetric candidate expansion with uncertainty-aware refinement to effectively handle sensor noise and sparse correspondences.

**What are the implications of the main findings?**

- The framework provides a plug-and-play solution for real-time robotics perception, bridging the gap between theoretical accuracy and practical deployment on low-power hardware.
- The results validate that optimized geometric methods remain superior in interpretability and generalization for industrial LiDAR sensing under challenging low-overlap conditions.

**Abstract:**

Accurate LiDAR point cloud registration on resource-constrained edge platforms is a prerequisite for intelligent robotics and industrial automation, yet it remains challenging because low-overlap matching, false correspondences, and fine alignment must be handled under limited computing budgets without GPU acceleration. While learning-based methods have advanced the field, their heavy hardware dependency and training requirements often hinder their practical deployment on mobile edge devices. To bridge this gap, this paper proposes GeoRescue, a training-free geometric registration framework designed for high-precision perception under stringent hardware limits. The method consists of three modular stages: Asymmetric Correspondence Expansion (ACE), which enlarges the candidate correspondence set to reduce the loss of true matches; Dynamic Geometric Topology Gating (DGTG), which suppresses false matches through distance-consistency-based hypothesis filtering; and Uncertainty-Aware Manifold Refinement (UAMR), which improves fine alignment by explicitly modeling local anisotropic noise via covariance-guided optimization. Experiments on 3DMatch, 3DLoMatch, and KITTI show that GeoRescue achieves registration recall rates of 84.84% and 41.27%, respectively, and a 94.95% success rate on KITTI. Remarkably, the framework matches the accuracy of high-capacity learning models while running on a GPU-free, 15 W edge CPU platform (Intel Core i5-8265U). These results indicate that GeoRescue provides a deployment-ready solution with an optimal efficiency–accuracy trade-off for LiDAR sensing and robotics perception in complex, real-world scenarios.

## 1. Introduction

LiDAR point cloud registration is a core component of robotics perception [1], mapping [2], autonomous navigation [3], and edge sensing systems [4]. By estimating the rigid transformation between partially overlapping point clouds, registration supports downstream tasks such as pose estimation, local map construction, scene reconstruction, and multi-frame fusion. In practical sensing systems, especially those deployed on mobile robots, unmanned platforms, and embedded devices, registration must remain accurate, robust, and efficient under limited power, memory, and runtime budgets. These requirements make registration particularly challenging on resource-constrained edge platforms, where GPU acceleration may be unavailable.

Classical geometric registration methods remain attractive in such settings because they are training-free like ICP [5], interpretable like RANSAC [6], and lightweight. However, their performance often degrades in low-overlap, noisy, and outlier-rich scenarios, where true correspondences are easily lost [7] and extreme false matches can severely affect transformation estimation [8]. In contrast, recent learning-based methods have achieved strong benchmark performance by learning more robust point features [9] and correspondence priors [10], but they usually require training data [11], larger models [12], and stronger hardware support. As a result, a practical gap still exists between high-performing registration methods and deployment-oriented solutions for computation-limited platforms.

Beyond direct pairwise LiDAR registration, multi-view camera and multi-scan systems provide another important route for handling low-overlap geometric perception. By observing the same scene from multiple viewpoints, such systems can introduce spatial redundancy, intermediate overlap relations, and global consistency constraints that are unavailable in a single source-target pair. However, these advantages usually rely on multiple calibrated or temporally connected observations and additional global optimization. In contrast, this work focuses on the more constrained pairwise LiDAR registration setting, where only one source point cloud and one target point cloud are available under resource-constrained edge conditions.

This gap motivates the development of a training-free geometric framework that preserves the deployability of classical methods while improving robustness under hardware limits. Rather than pursuing hardware-intensive feature learning, this work focuses on establishing a practical efficiency–accuracy trade-off for LiDAR point cloud registration on resource-constrained edge platforms. The main challenge is to improve correspondence preservation under low overlap, suppress false matches during transformation estimation, and refine local alignment without introducing training overhead or excessive computational burden.

To address these issues, this paper proposes a training-free geometric LiDAR point cloud registration framework, termed GeoRescue, which is composed of three stages: Asymmetric Correspondence Expansion (ACE), Dynamic Geometric Topology Gating (DGTG), and Uncertainty-Aware Manifold Refinement (UAMR). ACE enlarges the candidate correspondence set to reduce the loss of true matches in low-overlap conditions. DGTG suppresses unreliable correspondences through distance-consistency-based geometric filtering to improve transformation robustness. UAMR performs covariance-guided local refinement to enhance fine alignment quality. The overall framework follows a coarse-to-fine geometric pipeline and is designed for practical deployment on GPU-free and computation-limited platforms.

The aim of this study is to provide a deployment-oriented alternative to training-dependent registration methods for robotics perception and edge sensing scenarios. The proposed framework is evaluated on 3DMatch, 3DLoMatch, and KITTI, which are widely used benchmark datasets in 3D perception and LiDAR registration research. Experimental results show that the proposed method achieves competitive registration performance while maintaining favorable efficiency–accuracy trade-offs on a GPU-free 15 W edge platform. These results indicate that training-free geometric registration remains a practical solution for computation-limited LiDAR sensing applications.

The main contributions of this work are summarized as follows:

1. A training-free geometric LiDAR point cloud registration framework is developed for resource-constrained edge platforms.
2. An ACE–DGTG coarse matching strategy is introduced to improve candidate preservation and false-match suppression in low-overlap scenarios.
3. A UAMR module is designed to enhance fine alignment through covariance-guided uncertainty-aware refinement.
4. Extensive experiments on 3DMatch, 3DLoMatch, and KITTI demonstrate the practical value of the proposed method for robotics perception and edge sensing under computation-limited conditions.

## 2. Related Work

### 2.1. Classical Geometric Point Cloud Registration

Classical geometric point cloud registration methods remain an important foundation for 3D perception and LiDAR sensing. These methods usually rely on handcrafted descriptors [13], nearest-neighbor correspondence search, robust hypothesis generation, and local refinement [14]. Their main advantages are interpretability, low deployment cost, and the absence of a training stage, which make them attractive for resource-constrained platforms. In particular, descriptor-based matching and geometric verification pipelines are still widely used in practical systems because they can be implemented efficiently and adapted to different sensing conditions without retraining.

Despite these advantages, classical geometric methods often face clear limitations in low-overlap and outlier-rich scenarios. When overlap decreases, the number of reliable correspondences is reduced, while the proportion of ambiguous or false matches increases significantly. Under such conditions, descriptor-based nearest-neighbor matching can easily miss true correspondences, and transformation estimation becomes sensitive to hypothesis quality and local minima. Several recent studies in Sensors have shown that geometric registration can still be improved through better keypoint selection [15,16], geometric filtering [17,18] or weighted refinement [19], indicating that this research line remains active and relevant for practical sensing applications. At the same time, these studies also suggest that robustness under difficult overlap conditions is still a major challenge for lightweight geometric pipelines [20].

### 2.2. Learning-Based Point Cloud Registration

Learning-based registration methods have substantially advanced the field by improving feature representation [21], geometric consistency modeling [22], and using deep architectures to estimate correspondence reliability [23,24]. In particular, methods designed for low-overlap registration have shown clear advantages [25,26] in challenging scenarios where handcrafted descriptors and conventional matching strategies often degrade.

However, these improvements are commonly accompanied by increased dependence on model generalization [27]. Compared with classical geometric pipelines, learning-based methods usually require additional training stages, higher memory consumption due to cost volumes [28] and greater inference cost from large-scale transformers [29]. From the perspective of deployment-oriented sensing systems, especially on edge devices or low-power platforms, these requirements can become a practical constraint. Recent Sensors papers show that learning-based registration can achieve strong benchmark performance when supported by complete model design, ablation studies [30], and complexity analysis [31], but they also highlight the importance of robustness, computational cost, and experimental transparency when evaluating such methods. This indicates that benchmark accuracy alone is not sufficient for deployment-oriented registration, particularly when hardware constraints are part of the problem setting.

### 2.3. Low-Overlap Registration and Resource-Constrained Deployment

Low-overlap registration remains one of the most difficult settings in point cloud alignment. As overlap decreases, true correspondences become extremely sparse [32,33], the risk of mismatch increases significantly [34], and rigid transformation estimation becomes less stable. This difficulty is reflected in both geometric and learning-based methods, although the underlying failure mechanisms differ. For geometric methods, the main issue is often the loss of true matches and the insufficient rejection of false hypotheses. For learning-based methods, performance can still depend on the learned representation, the training domain, and the computational budget available at inference time.

Compared with the extensive literature on improving registration accuracy, relatively fewer studies focus explicitly on hardware-accelerated or FPGA-limited deployment [35]. In practical robotics perception and edge sensing scenarios, the problem is not only how to obtain a correct transformation, but also how to do so using limited computing resources, low power consumption, and reduced implementation complexity. Recent Sensors articles suggest that the journal values methods that combine algorithmic improvement with practical engineering significance, including robustness analysis, complexity analysis, and deployment-related discussion. This creates a clear research space for methods that are training-free, geometrically grounded, and explicitly designed for resource-constrained platforms rather than only for peak benchmark performance.

### 2.4. Multi-View Vision and Multi-View Registration Context

Multi-view camera and multi-scan systems provide a practical complementary mechanism for low-overlap point cloud registration. In a single-pair setting, registration depends only on one source point cloud and one target point cloud; when their overlap is limited, reliable correspondences become sparse and transformation estimation becomes sensitive to false matches. In contrast, multi-view systems observe the same scene from different viewpoints and can provide additional spatial redundancy, intermediate overlap relations, and more reliable geometric constraints. For example, multi-camera triangulation can recover more stable 3D geometric information from common features observed across views [36], while joint selection of camera orientations and feature projections can help retain more informative geometric cues before registration [37]. Therefore, in practical LiDAR or camera-LiDAR systems, multi-view techniques can be used as an upstream assistance module to select more reliable views, scan pairs, or feature regions before applying the proposed pairwise registration method.

The proposed GeoRescue framework and multi-view registration are therefore complementary rather than competing. GeoRescue strengthens the pairwise registration step by improving candidate correspondence preservation, false-match suppression, and uncertainty-aware refinement, while multi-view optimization can further improve global accuracy when multiple observations are available. For instance, bundle adjustment can refine multiple transformations jointly to improve global consistency [38], pose-graph initialization and history reweighting can reduce the influence of unreliable scan-pair relations [39], and overlap-confidence estimation can help identify reliable scan connections in unknown-overlap multiview registration [40]. Thus, GeoRescue can serve as a robust pairwise front-end in a larger multi-view pipeline, providing cleaner relative transformations before global pose-graph or bundle-adjustment refinement. In practical multi-angle sensing applications, combining GeoRescue with multi-view selection and global optimization can provide additional spatial redundancy and consistency constraints, thereby improving registration accuracy and robustness beyond pairwise registration alone.

### 2.5. Summary of the Research Gap

In summary, existing geometric registration methods remain attractive for computation-limited deployment because they are lightweight, interpretable, and training-free, but they often lack sufficient robustness under low-overlap and high-outlier conditions. Learning-based methods have improved performance in challenging benchmark scenarios, yet their training dependency and hardware cost can limit their applicability on low-power edge platforms. Therefore, a practical gap still exists between high-performing registration methods and deployment-oriented solutions for resource-constrained sensing systems.

Motivated by this gap, the present work develops a training-free geometric LiDAR registration framework for resource-constrained edge platforms. The proposed method focuses on three closely related issues: preserving candidate correspondences under low overlap, suppressing false matches during transformation estimation, and improving fine alignment without introducing heavy training or inference overhead. In this sense, the method is intended not as a replacement for all high-capacity learning-based approaches, but as a deployment-oriented alternative for robotics perception and edge sensing scenarios where hardware limits are a central concern.

## 3. Materials and Methods

### 3.1. Overview of the Proposed Framework

This study proposes GeoRescue, a training-free geometric LiDAR point cloud registration framework for resource-constrained edge platforms. The framework is intended for robotics perception and edge sensing scenarios in which GPU acceleration may be unavailable and the available power, memory, and runtime budgets are strictly limited. To address these constraints, the proposed method adopts a coarse-to-fine geometric pipeline composed of three stages: Asymmetric Correspondence Expansion (ACE), Dynamic Geometric Topology Gating (DGTG), and Uncertainty-Aware Manifold Refinement (UAMR).

The proposed framework takes a source point cloud and a target point cloud as input and estimates the rigid transformation between them without model training. Instead of relying on hardware-intensive learned registration pipelines, the method focuses on candidate expansion in feature space, geometric hypothesis filtering, and uncertainty-aware local refinement using lightweight geometric operations. This design makes the framework suitable for deployment-oriented LiDAR registration under hardware limits.

In the first stage, ACE enlarges the candidate correspondence pool to reduce the loss of true matches under low-overlap conditions. In the second stage, DGTG evaluates the geometric consistency of candidate hypotheses and removes unreliable matches before rigid transformation estimation. In the final stage, UAMR performs covariance-guided refinement on the transformation manifold to improve fine alignment quality. Through this three-stage design, the registration problem is converted into a progressively constrained geometric estimation process that is more robust to low overlap, mismatch, and limited computational resources.

The proposed framework is designed to provide a practical efficiency–accuracy trade-off for LiDAR registration on low-power edge platforms. Accordingly, its overall design emphasizes three properties: training-free operation, geometric interpretability, and deployment feasibility. These properties distinguish the framework from training-dependent registration pipelines that usually require larger models, stronger hardware support, and additional inference overhead.

**Figure 1:**
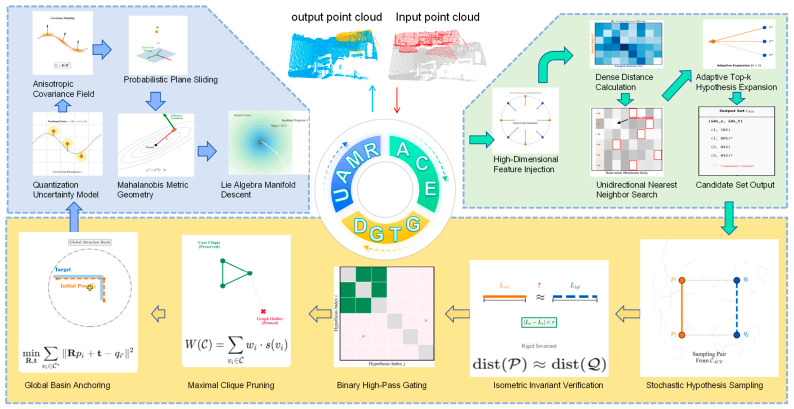
Overall pipeline of the proposed training-free geometric registration framework. ACE generates an expanded candidate set C_ACE through bidirectional nearest-neighbor search and adaptive Top-K expansion, where (*) denotes expanded or collision candidates; DGTG filters hypotheses via isometric invariant verification and binary gating, where red boxes indicate gated inliers and × marks rejected outliers; UAMR performs covariance-guided refinement on the transformation manifold. Legend: Blue/orange/gray blocks represent input/output point clouds, feature tensors, and computation stacks; yellow/green/blue arrows indicate ACE/DGTG/UAMR flows; ‘NN’, ‘min’, and ‘argsort’ denote nearest-neighbor retrieval, pooling, and sorting, respectively.

As shown in Figure 1, the overall workflow consists of three sequential modules. ACE generates an expanded candidate correspondence set from the input point clouds, DGTG filters geometrically inconsistent hypotheses to support robust coarse transformation estimation, and UAMR refines the coarse result through uncertainty-aware local optimization. The final output is the rigid transformation that aligns the source point cloud to the target point cloud.

The benchmark datasets, edge hardware platform, mathematical formulation, and the three main modules of the framework are described in the following subsections.

### 3.2. Datasets and Edge Platform

#### 3.2.1. Public Benchmark Datasets

The proposed method was evaluated on three public datasets: 3DMatch, 3DLoMatch, and KITTI. These datasets were selected because they are widely used benchmarks in 3D perception, LiDAR registration, and autonomous sensing research, and they provide a common basis for comparison with recent registration methods.

3DMatch is a standard indoor point cloud registration benchmark built from RGB-D reconstructions [41]. It is commonly used to evaluate registration performance under partial overlap, sensor noise, and scene complexity. 3DLoMatch is a more challenging subset derived from 3DMatch, in which overlap ratios are significantly lower and correspondence ambiguity is substantially increased. KITTI is a standard outdoor dataset for autonomous driving and LiDAR-based perception [42], and it is widely used to evaluate registration and pose estimation in large-scale outdoor sensing environments.

In this work, 3DMatch and 3DLoMatch were used to evaluate indoor low-overlap registration performance, while KITTI was used to assess outdoor LiDAR registration behavior under increasing frame-gap difficulty. The use of these datasets is consistent with current point cloud registration research in robotics perception and LiDAR sensing. Their adoption in this study is intended to ensure comparability with existing registration literature and to support a deployment-oriented evaluation under representative benchmark conditions.

#### 3.2.2. Edge Hardware Platform

To examine practical deployment feasibility under hardware limits, the proposed GeoRescue framework was implemented and tested on a GPU-free edge platform equipped with an Intel Core i5-8265U processor (Intel Corp., Santa Clara, CA, USA) and 20.0 GB of RAM. The system operated on the Windows 10 operating system (Microsoft Corp., Redmond, WA, USA).

The registration algorithm was developed using the Python programming language (version 3.10.20; Python Software Foundation, Wilmington, DE, USA). All point cloud processing, normal estimation, and feature calculations were conducted using the Open3D library (version 0.19.0; Intel Corp., Santa Clara, CA, USA). Numerical matrix operations and spatial KD-Tree searches were supported by NumPy (version 2.2.6; NumFOCUS, Austin, TX, USA) and SciPy (version 1.15.3; NumFOCUS, Austin, TX, USA). The source code and experimental configurations are available online at https://github.com/ZK131/GeoRescue (accessed on 25 May 2026).

### 3.3. Asymmetric Correspondence Expansion (ACE)

Let the source and target point clouds be denoted by

(1) $$P=\{p_{i}{\}}_{i=1}^{N},Q=\{q_{j}{\}}_{j=1}^{M},$$

where $p_{i},q_{j}\in R^{3}$. The objective of rigid point cloud registration is to estimate a transformation T that aligns P to Q, where

(2) T(x)=Rx+t,

with R∈SO(3) and $t\in R^{3}$. Given a candidate correspondence set C, the classical rigid registration objective can be written as

(3) $$\underset{R,t}{min}\;\sum_{(i,j)\in C}\;\;\|Rp_{i}+t-q_{j}{\|}_{2}^{2}.$$

In low-overlap registration, however, the main difficulty often lies before the optimization in Equation (3). When overlap decreases, true correspondences become sparse, while ambiguous and false matches increase substantially. Under such conditions, one-way nearest-neighbor assignment may discard potentially valid correspondences at an early stage, which makes the subsequent geometric verification and transformation estimation unstable. This issue is particularly critical on resource-constrained edge platforms, where exhaustive matching or heavy learned refinement is undesirable. Unlike recent continuous state-space models designed for long-sequence point clouds [43,44] or cross-modal matching pipelines [45], the proposed ACE module operates on lightweight handcrafted geometric descriptors computed from the input point clouds, rather than learned features, to ensure efficiency.

To alleviate this problem, the proposed Asymmetric Correspondence Expansion (ACE) module enlarges the candidate correspondence pool through bidirectional feature-space matching and adaptive candidate fusion. Let $F_{P}=\{f_{i}{\}}_{i=1}^{N}$ and $F_{Q}=\{g_{j}{\}}_{j=1}^{M}$ denote the FPFH descriptor sets extracted from the downsampled source and target point clouds, respectively. A pairwise feature distance matrix is computed as

(4) $$D_{ij}=\|f_{i}-g_{j}{\|}_{2}$$

Here, $D_{ij}$ measures the feature-space dissimilarity between source point $p_{i}$ and target point $q_{j}$. Smaller values of $D_{ij}$ indicate higher local feature similarity and therefore higher candidate matching confidence in feature space.

Based on the distance matrix, ACE first constructs a forward nearest-neighbor proposal set from the source cloud to the target cloud:

(5) $$C_{ACE,fwd}=\left\{\left(i,\underset{j}{argmin}D_{ij}\right)|i=1,\ldots ,N\right\}$$

It then constructs a backward nearest-neighbor proposal set from the target cloud to the source cloud:

(6) $$C_{ACE,bwd}=\left\{\left(\underset{i}{argmin}D_{ij},j\right)|j=1,\ldots ,M\right\}.$$

The bidirectional proposal mechanism reduces the asymmetry caused by overlap imbalance and local ambiguity. The expanded candidate set is therefore defined as

(7) $$C_{ACE}=C_{ACE,fwd}\cup C_{ACE,bwd}.$$

To further improve candidate coverage, an adaptive Top-K expansion strategy is adopted. For each source feature $f_{i}$, the K nearest target candidates are retained; similarly, for each target feature $g_{j}$, the K nearest source candidates are retained. The resulting Top-K expanded candidate set is written as

(8) $$C_{ACE}^{\left(K\right)}=\{(i,j)|j\in {TopK}_{Q}(i)\}\cup \{(i,j)|i\in {TopK}_{P}(j)\}.$$

The expansion factor K is treated as a tunable parameter rather than a learned or per-pair dynamically predicted value. A default value of K = 3 is used in all reported experiments, which is selected based on a comprehensive parameter sensitivity analysis presented in Section 4.6. This value provides the optimal balance between registration recall and computational cost on low-overlap benchmarks.

In practice, duplicate candidate pairs are removed after bidirectional fusion, and only valid candidate indices are retained. The output of ACE is not treated as the final inlier set. Instead, it is designed as a recall-oriented candidate pool for downstream geometric filtering. This role assignment is important for low-overlap edge deployment: ACE focuses on preserving potentially valid correspondences using lightweight operations, while later stages are responsible for rejecting false hypotheses and refining the transformation.

**Figure 2:**
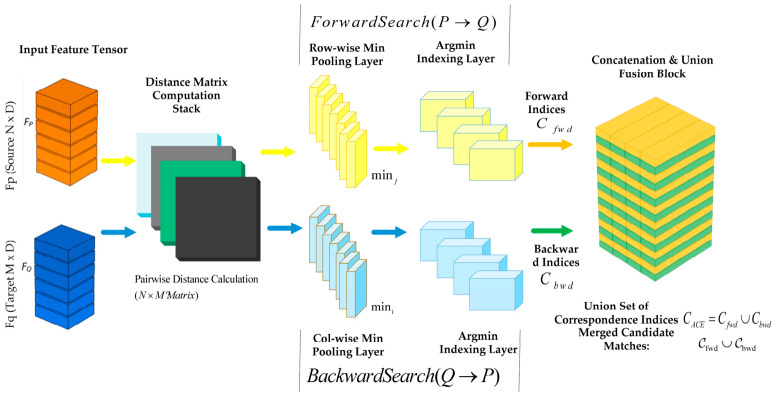
Implementation-oriented architecture of the Asymmetric Correspondence Expansion (ACE) module. Orange and blue input feature tensors represent the source ($F_{P}$) and target ($F_{Q}$) point cloud descriptors, respectively. Yellow arrows indicate the forward search flow (P→Q), blue arrows indicate the backward search flow (Q→P), and the green arrow denotes the concatenation and union fusion step. Yellow and light-blue intermediate blocks represent the forward indices ($C_{fwd}$) and backward indices ($C_{bwd}$), respectively. The final yellow-and-green stacked block represents the merged candidate correspondence set $C_{ACE}$.

Figure 2 illustrates the implementation-oriented structure of ACE. Source and target feature tensors are first used to construct a pairwise distance matrix, after which row-wise and column-wise candidate selection are performed to generate forward and backward proposal sets, respectively. The two directional proposal sets are then merged through a union operation to form the expanded candidate pool. This figure shows that ACE improves candidate preservation through lightweight bidirectional matching and fusion rather than training-dependent correspondence correction, which is consistent with the deployment-oriented design of the overall framework.

**Figure 3:**
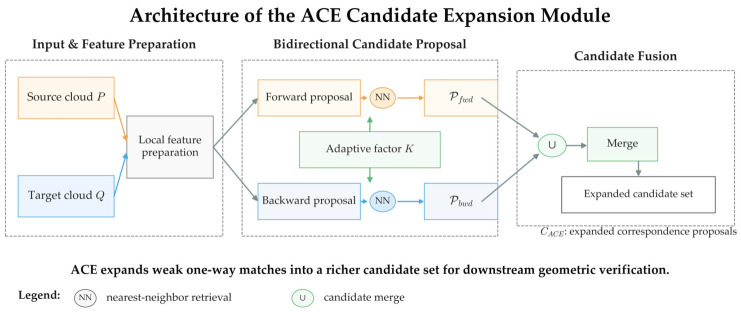
Architecture of the ACE Candidate Expansion Module. Orange arrows indicate the source point cloud (P) data flow through local feature preparation to the forward proposal branch; blue arrows indicate the target point cloud (Q) data flow to the backward proposal branch; green arrows represent the adaptive factor K control flow and the final merging stream into the expanded candidate set; and gray arrows denote the input of forward ($P_{fwd}$) and backward ($P_{bwd}$) proposal sets into the union (∪) fusion block. ACE expands weak one-way matches into a richer candidate set for downstream geometric verification. Legend: NN, nearest-neighbor retrieval; U, candidate merge.

Figure 3 provides a conceptual view of the ACE module. The figure highlights that ACE does not directly determine the final inlier set; instead, it expands weak one-way matching results into a richer candidate pool for downstream geometric verification. The adaptive factor K controls the coverage of the expanded set, while the final fusion step combines forward and backward proposals into a unified output. This role separation is important because ACE is designed to improve candidate recall, whereas the subsequent DGTG stage is responsible for geometric filtering and the later UAMR stage is responsible for local refinement.

For implementation transparency, the ACE procedure is summarized as follows in Algorithm 1.
**Algorithm 1:** Asymmetric Correspondence Expansion (ACE)$\mathrm{Input}:\;\mathrm{Source}\;\mathrm{feature}\;\mathrm{set}\;F_{P}$$,\;\mathrm{target}\;\mathrm{feature}\;\mathrm{set}\;F_{Q}$, expansion factor K $\mathrm{Output}:\;\mathrm{Expanded}\;\mathrm{candidate}\;\mathrm{correspondence}\;\mathrm{set}\;C_{ACE}$1Compute the pairwise feature distance matrix D$,\;\mathrm{where}\;D_{ij}=\|f_{i}-g_{j}{\|}_{2}$ (Equation (4)).2$\mathrm{Perform}\;\mathrm{forward}\;\mathrm{nearest}-\mathrm{neighbor}\;\mathrm{search}\;\mathrm{to}\;\mathrm{obtain}\;\mathrm{proposal}\;\mathrm{set}\;C_{ACE,fwd}$ (Equation (5)).2$\mathrm{Perform}\;\mathrm{backward}\;\mathrm{nearest}-\mathrm{neighbor}\;\mathrm{search}\;\mathrm{to}\;\mathrm{obtain}\;\mathrm{proposal}\;\mathrm{set}\;C_{ACE,bwd}$ (Equation (6)).3Retain the Top-K$\;\mathrm{nearest}\;\mathrm{candidates}\;\mathrm{in}\;\mathrm{both}\;\mathrm{directions}\;\mathrm{to}\;\mathrm{form}\;C_{ACE}^{\left(K\right)}$ (Equation (8)).4Merge the forward, backward, and Top-K$\;\mathrm{proposal}\;\mathrm{sets}\;\mathrm{to}\;\mathrm{construct}\;C_{ACE}$ (Equation (7)).5Remove duplicate correspondence pairs and retain valid candidate indices.6$\mathrm{return}\;C_{ACE}$

### 3.4. Dynamic Geometric Topology Gating (DGTG)

Although the ACE module improves candidate preservation under low-overlap conditions, the expanded candidate pool still contains ambiguous and false correspondences. If these candidates are passed directly to rigid transformation estimation, the resulting solution can become unstable, especially when outliers dominate the candidate set. This issue is more severe on resource-constrained edge platforms, where computationally heavy global verification is undesirable. To address this problem, the proposed Dynamic Geometric Topology Gating (DGTG) module performs lightweight geometric filtering before coarse transformation estimation.

The key idea of DGTG is that valid correspondences should satisfy pairwise geometric consistency under a rigid transformation. Let $h_{m}=(i,j)$ and $h_{n}=(i^{\prime },j^{\prime })$ denote two candidate correspondences selected from $C_{ACE}$. If both correspondences are consistent with the same rigid motion, then the Euclidean distance between the two source points should be close to the Euclidean distance between the corresponding target points. Based on this principle, a pairwise geometric residual is defined as

(9) $$r_{mn}=\left|\|p_{i}-p_{i^{\prime }}{\|}_{2}-\|q_{j}-q_{j^{\prime }}{\|}_{2}\right|.$$

Here, $r_{mn}$ measures the deviation of distance preservation between two candidate correspondences. Smaller values of $r_{mn}$ indicate stronger geometric compatibility.

A binary geometric gate is then introduced to determine whether a candidate pair satisfies the distance-consistency constraint:

(10) $$g_{mn}=1(r_{mn}<\tau ).$$

In Equation (10), τ denotes the geometric consistency threshold and 1(⋅) is the indicator function. If $g_{mn}=1$, the two candidate correspondences are regarded as mutually compatible; otherwise, they are treated as inconsistent. While recent robust estimators seek maximal cliques on correspondence graphs [46,47] or extract dynamic neighborhood features [48], DGTG provides a much lighter pairwise gating mechanism tailored for edge CPU execution.

**Figure 4:**
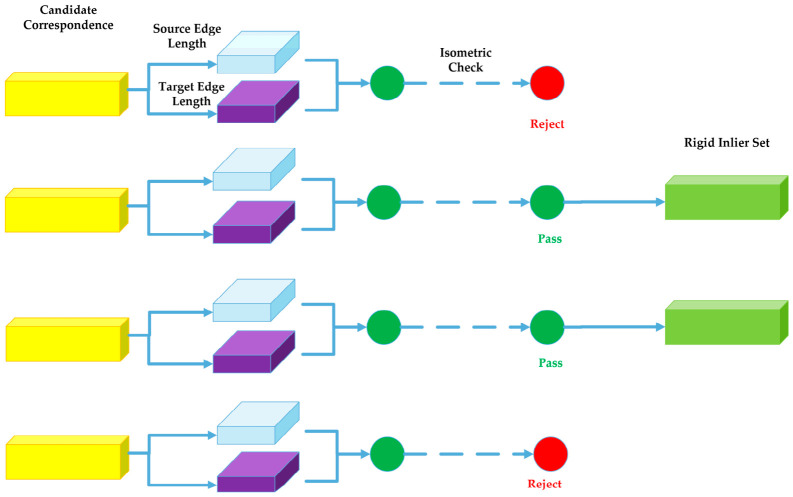
Conceptual workflow of geometric consistency checking in the DGTG module.

Figure 4 illustrates the core acceptance mechanism of DGTG. Each candidate correspondence is examined through source-side and target-side edge-length comparison under an isometric consistency assumption. Candidates satisfying the geometric constraint are retained as rigid-compatible hypotheses, whereas inconsistent candidates are rejected by the binary gate. This figure emphasizes that DGTG improves candidate precision after the recall-oriented ACE stage and provides a cleaner input for coarse transformation estimation.

Based on the binary gate, each candidate correspondence is assigned a voting score that reflects how many other candidates are geometrically compatible with it:

(11) $$v_{m}=\sum_{n\neq m}\;\;g_{mn}.$$

A larger value of $v_{m}$ indicates that candidate $h_{m}$ is supported by more geometrically consistent neighbors and is therefore more likely to belong to the rigid-consistent inlier structure. The candidate hypotheses are then ranked in descending order according to their voting scores:

(12) $$\pi =argsort\left(\left\{v_{m}\right\}\right).$$

Based on the ranking result, DGTG constructs a filtered inlier candidate set by retaining only hypotheses whose geometric support exceeds a gating threshold γ:

(13) $$I_{DGTG}=\left\{h_{m}\in C_{ACE}|v_{m}\geq \gamma \right\}.$$

The output set $I_{DGTG}$ is used as the input to coarse transformation estimation. In this way, DGTG suppresses geometrically inconsistent hypotheses before rigid estimation, which improves robustness without introducing learned outlier rejection or computationally expensive exhaustive verification.

For deployment-oriented registration, it is also important to avoid unnecessary sampling overhead. Therefore, the inlier ratio estimated after DGTG filtering is used to guide the subsequent robust estimation schedule. Let the estimated inlier ratio be

(14) $$\overset{ˆ}{r}=\frac{\left|I_{DGTG}\right|}{\left|C_{ACE}\right|}.$$

Given a confidence level η and a minimal sample size s, the required number of RANSAC iterations is adaptively estimated as

(15) $$N_{iter}=\left\lceil \frac{log(1-\eta )}{log(1-{\overset{ˆ}{r}}^{s})}\right\rceil .$$

Equation (15) allows the hypothesis evaluation process to adapt to candidate quality after DGTG filtering. When the filtered candidate pool is cleaner, fewer iterations are required; when the pool remains noisy, the iteration budget is increased accordingly. This adaptive mechanism is useful on low-power hardware because it helps balance robustness and runtime cost.

In practice, the parameters introduced in this stage are physically grounded and do not require heavy per-scene tuning. The minimal sample size s is strictly set to 3, which is the mathematical minimum required to compute a rigid 3D transformation. The confidence level η is set to 0.999, as is standard in robust estimation.

To ensure generalization across datasets with different scales and point densities, the geometric consistency threshold τ is scaled relative to the voxel downsampling size (e.g., τ=2×voxel_size). This naturally accounts for spatial discretization errors. For instance, τ is 0.1 m for indoor datasets (voxel size = 0.05 m) and is scaled up accordingly for large-scale outdoor datasets. Furthermore, instead of assigning a rigid, absolute scalar value for the gating threshold γ, we implement a soft-ranking mechanism. The candidates are sorted by their votes, and γ is dynamically determined to retain the top 30% (i.e., elite ratio = 0.3) of the highest-voted hypotheses. This dynamic thresholding ensures that DGTG robustly extracts a high-quality elite subset regardless of the absolute number of correspondences or specific environmental structures.

**Figure 5:**
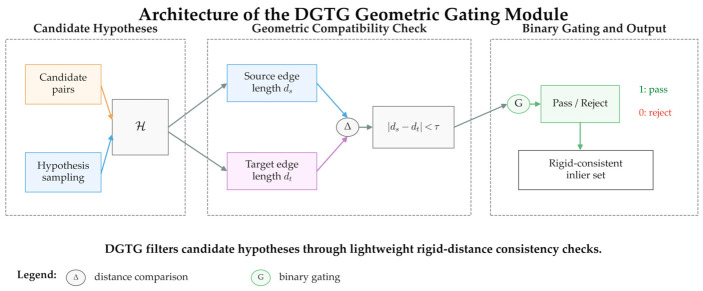
Implementation-oriented architecture of the Dynamic Geometric Topology Gating (DGTG) module.

Figure 5 presents the implementation-oriented structure of DGTG. Candidate correspondences generated by ACE are first converted into hypothesis pairs. Pairwise source-side and target-side edge lengths are then compared to evaluate rigid distance consistency. The resulting compatibility judgments are accumulated through geometric voting, after which unreliable hypotheses are suppressed before coarse transformation estimation. This figure shows that DGTG improves robustness through lightweight geometric filtering rather than training-dependent confidence learning, which is consistent with the resource-constrained design of the proposed framework.

For implementation transparency, the DGTG procedure is summarized in Algorithm 2.
**Algorithm 2:** Dynamic Geometric Topology Gating (DGTG)$\mathrm{Input}:\;\mathrm{Expanded}\;\mathrm{candidate}\;\mathrm{set}\;C_{ACE}$, source point cloud P, target point cloud Q, geometric threshold τ, support threshold γ $\mathrm{Output}:\;\mathrm{Filtered}\;\mathrm{inlier}\;\mathrm{candidate}\;\mathrm{set}\;I_{DGTG}$$,\;\mathrm{adaptive}\;\mathrm{iteration}\;\mathrm{number}\;N_{iter}$1$\mathrm{Initialize}\;\mathrm{voting}\;\mathrm{scores}\;v_{m}\leftarrow 0$ for all hypotheses.2$\mathrm{for}\;\mathrm{each}\;\mathrm{candidate}\;\mathrm{hypothesis}\;\mathrm{pair}\;h_{m}=(i,j)$$\;\mathrm{and}\;h_{n}=(i^{\prime },j^{\prime })$$\;\mathrm{sampled}\;\mathrm{from}\;C_{ACE}$ do3$\mathrm{Compute}\;\mathrm{pairwise}\;\mathrm{geometric}\;\mathrm{residual}\;r_{mn}=|\|p_{i}-p_{i^{\prime }}{\|}_{2}-\|q_{j}-q_{j^{\prime }}{\|}_{2}|$ (Equation (9)).4$\mathrm{Evaluate}\;\mathrm{the}\;\mathrm{binary}\;\mathrm{geometric}\;\mathrm{gate}\;g_{mn}=1(r_{mn}<\tau )$ (Equation (10)).5$\mathrm{Accumulate}\;\mathrm{voting}\;\mathrm{score}\;\mathrm{for}\;\mathrm{the}\;\mathrm{hypothesis}:\;v_{m}\leftarrow v_{m}+g_{mn}$ (Equation (11)).6end for7$\mathrm{Rank}\;\mathrm{candidate}\;\mathrm{hypotheses}\;\mathrm{in}\;\mathrm{descending}\;\mathrm{order}:\;\pi =\mathrm{argsort}(\{v_{m}\})$ (Equation (12)).8$\mathrm{Retain}\;\mathrm{geometrically}\;\mathrm{supported}\;\mathrm{candidates}:\;I_{DGTG}=\{h_{m}\in C_{ACE}|v_{m}\geq \gamma \}$ (Equation (13)).9$\mathrm{Estimate}\;\mathrm{the}\;\mathrm{inlier}\;\mathrm{ratio}\;\hat{r}=|I_{DGTG}|/|C_{ACE}|$ (Equation (14)).10$\mathrm{Compute}\;\mathrm{the}\;\mathrm{adaptive}\;\mathrm{iteration}\;\mathrm{number}\;N_{iter}=\frac{log(1-\eta )}{log(1-{\hat{r}}^{s})}$ (Equation (15)).11$\mathrm{return}\;I_{DGTG}$$\;\mathrm{and}\;N_{iter}$

### 3.5. Uncertainty-Aware Manifold Refinement (UAMR)

After the DGTG stage, the retained candidate set $I_{DGTG}$ provides a more reliable basis for coarse rigid transformation estimation. However, even after geometric filtering, residual local misalignment may still remain because low-overlap matching, sensor noise, and point discretization can affect the quality of the coarse solution. This issue is particularly relevant in deployment-oriented LiDAR sensing, where lightweight coarse estimation alone may be insufficient for obtaining stable fine alignment. To improve the final transformation quality without introducing heavy model inference or training-dependent refinement, the proposed Uncertainty-Aware Manifold Refinement (UAMR) module performs covariance-guided local optimization on the transformation manifold.

The core idea of UAMR is that different correspondences should not be treated equally during refinement. In practical point cloud registration, local geometric quality can vary considerably across points due to anisotropic sampling, surface structure, and measurement uncertainty. Therefore, the refinement stage should account for local geometric reliability instead of using only isotropic point-to-point residual minimization.

For each inlier correspondence, a local covariance matrix is estimated from the neighborhood of the source point. Let $N(p_{i})$ denote the local neighborhood of point $p_{i}$, and let ${\overset{‾}{p}}_{i}$ denote the neighborhood centroid. The covariance matrix is defined as

(16) $$\Sigma_{i}=\frac{1}{\left|N(p_{i})\right|}\sum_{p\in N(p_{i})}\;\;(p-{\overset{‾}{p}}_{i})(p-{\overset{‾}{p}}_{i}{)}^{⊤}+\epsilon I.$$

In Equation (16), ϵI is a small regularization term added to improve numerical stability. The local neighborhood $N(p_{i})$ is defined using a hybrid radius-based nearest-neighbor search. To ensure robustness across varying point densities while maintaining computational efficiency, the search radius is set to twice the voxel downsampling size (e.g., 0.1 m for the indoor setting) with a maximum limit of 30 nearest neighbors. The covariance matrix Σi characterizes the local anisotropic structure around point pi, which is later used to weight the refinement residuals.

To model the joint uncertainty of a correspondence pair $\left(p_{i},q_{j}\right)$, the covariance of the transformed source point and the target point is combined as

(17) $$\Sigma_{ij}=R\Sigma_{i}R^{⊤}+\Sigma_{j}.$$

Equation (17) propagates the source-side covariance into the target frame and merges it with the target-side covariance. In this way, the local geometric reliability of both sides is incorporated into the refinement process. This targeted statistical optimization avoids the computational overhead of self-supervised neural calibration [49] or generative pre-training [50] during the fine alignment phase.

**Figure 6:**
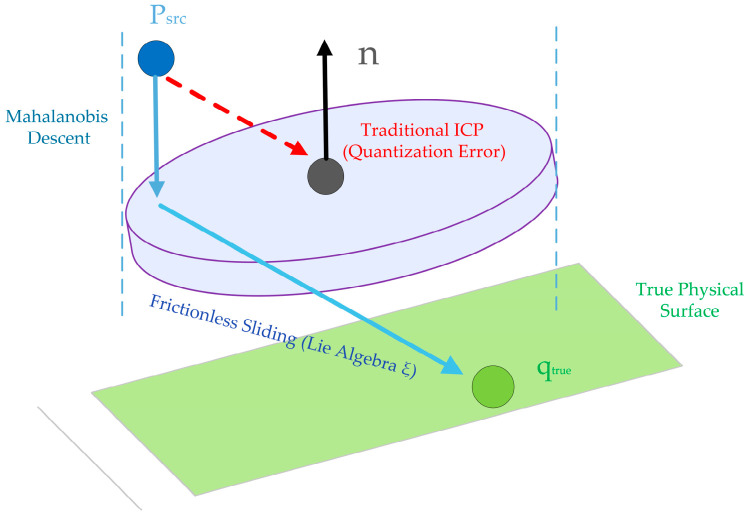
Conceptual illustration of uncertainty-aware local modeling in the UAMR module. The blue solid arrow indicates the Mahalanobis descent direction; the blue dashed line denotes the vertical projection axis of source point $p_{src}$ onto the manifold; the red dashed arrow represents the traditional ICP quantization error; the black arrow n indicates the surface normal; the light-blue arrow shows the frictionless sliding trajectory along the Lie algebra ξ toward the true point $q_{true}$; and the green plane represents the true physical surface.

Figure 6 conceptually illustrates the motivation of UAMR. Different local regions may exhibit different geometric reliability, and the uncertainty associated with a correspondence is generally anisotropic rather than isotropic. This means that some residual directions are more trustworthy than others during local alignment. By explicitly modeling such local uncertainty, UAMR avoids treating all residual components equally and provides a more stable basis for fine refinement under noisy and low-overlap conditions.

Based on the joint covariance, the refinement objective is written as a Mahalanobis-weighted residual minimization problem:

(18) $$E_{UAMR}(R,t)=\sum_{(i,j)\in I_{DGTG}}\;\;(Rp_{i}+t-q_{j}{)}^{⊤}\Sigma_{ij}^{-1}(Rp_{i}+t-q_{j}).$$

Compared with ordinary Euclidean residual minimization, Equation (18) assigns different weights to residual directions according to local uncertainty. This is beneficial in anisotropic or locally ambiguous regions because it reduces the influence of unreliable directions while preserving informative geometric structure.

To avoid directly optimizing the transformation in Euclidean parameter space, UAMR performs iterative updates on the Lie algebra manifold. Let $T_{k}$ denote the current transformation estimate at iteration k, and let $\xi_{k}\in se(3)$ denote the incremental twist vector. The transformation update is expressed as

(19) $$T_{k+1}=exp({\overset{ˆ}{\xi }}_{k})T_{k}.$$

Here, ${\overset{ˆ}{\xi }}_{k}$ is the matrix form of the twist vector and exp(⋅) denotes the matrix exponential map from the Lie algebra to the rigid transformation group. This formulation allows the refinement process to remain on the valid rigid-motion manifold during optimization.

The iteration is terminated when the update magnitude becomes sufficiently small or when the objective improvement falls below a preset threshold. The convergence criterion is expressed as

(20) $$\|\xi_{k}{\|}_{2}<\varepsilon \mathrm{or}|E_{k+1}-E_{k}|<\varepsilon .$$

Equation (20) prevents unnecessary refinement iterations and helps preserve efficiency on computation-limited platforms. This is particularly important for the present study, which aims to maintain a favorable efficiency–accuracy trade-off under hardware limits.

**Figure 7:**
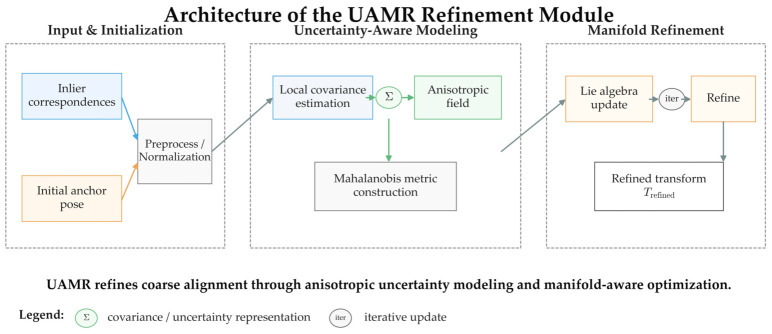
Implementation-oriented architecture of the Uncertainty-Aware Manifold Refinement (UAMR) module.

Figure 7 shows the implementation-oriented structure of the UAMR module. Filtered inlier correspondences and an initial coarse transformation are first used as input. Local covariance estimation is then performed to construct an anisotropic uncertainty model, from which a Mahalanobis metric is derived for refinement. The transformation is subsequently updated on the manifold through iterative local optimization. This figure emphasizes that UAMR improves fine alignment through lightweight geometric modeling rather than additional learned refinement, which is consistent with the resource-constrained design of the proposed framework.

For implementation transparency, the UAMR procedure is summarized in Algorithm 3.
**Algorithm 3:** Uncertainty-Aware Manifold Refinement (UAMR)$\mathrm{Input}:\;\mathrm{Filtered}\;\mathrm{inlier}\;\mathrm{candidate}\;\mathrm{set}\;I_{DGTG}$$,\;\mathrm{coarse}\;\mathrm{rigid}\;\mathrm{transformation}\;T_{0}$, source point cloud P, target point cloud Q Output: Refined rigid transformation T1$\mathrm{Initialize}\;\mathrm{the}\;\mathrm{transformation}\;\mathrm{estimate}\;\mathrm{as}\;T\leftarrow T_{0}$.2$\mathrm{for}\;\mathrm{each}\;\mathrm{corresponding}\;\mathrm{point}\;\mathrm{pair}\;\mathrm{in}\;I_{DGTG}$ do3$\mathrm{Estimate}\;\mathrm{local}\;\mathrm{covariance}\;\mathrm{matrices}\;\Sigma_{i}$$\;\mathrm{and}\;\Sigma_{j}$ for the source and target neighborhoods (Equation (16)).4end for5Repeat6$\mathrm{for}\;\mathrm{each}\;\mathrm{correspondence}\;(i,j)\in I_{DGTG}$ do7$\mathrm{Construct}\;\mathrm{joint}\;\mathrm{correspondence}\;\mathrm{covariance}\;\Sigma_{ij}=R\Sigma_{i}R^{⊤}+\Sigma_{j}$ (Equation (17)).8end for9$\mathrm{Form}\;\mathrm{the}\;\mathrm{Mahalanobis}-\mathrm{weighted}\;\mathrm{refinement}\;\mathrm{objective}\;E_{UAMR}(R,t)$ (Equation (18)).10$\mathrm{Compute}\;\mathrm{the}\;\mathrm{manifold}\;\mathrm{update}\;{\hat{\xi }}_{k}$$\;\mathrm{and}\;\mathrm{update}\;\mathrm{current}\;\mathrm{transformation}:\;T_{k+1}=exp({\hat{\xi }}_{k})T_{k}$ (Equation (19)).11$\mathrm{Check}\;\mathrm{the}\;\mathrm{convergence}\;\mathrm{condition}:\;\|\xi_{k}{\|}_{2}<\varepsilon$$\;\mathrm{or}\;|E_{k+1}-E_{k}|<\varepsilon$ (Equation (20)).12until convergence condition is met13return Refined rigid transformation T

The role of UAMR within the overall framework is different from those of ACE and DGTG. ACE is designed to preserve candidate correspondences, and DGTG is designed to improve candidate precision through geometric filtering. In contrast, UAMR is responsible for improving the final alignment quality after coarse rigid estimation. This separation of roles is important for the proposed training-free framework because it allows each stage to solve a specific subproblem using lightweight geometric operations, which is consistent with the deployment-oriented design philosophy of the method.

## 4. Results

### 4.1. Experimental Setup

#### 4.1.1. Evaluation Protocol

The proposed method was evaluated on 3DMatch, 3DLoMatch, and KITTI under the standard benchmark protocols of the corresponding datasets. 3DMatch and 3DLoMatch were used to assess indoor registration performance under partial-overlap conditions, while KITTI was used to evaluate outdoor LiDAR registration under different frame-gap difficulty settings. For all datasets, the core registration pipeline remained unchanged, and only dataset-specific preprocessing and evaluation configurations were adjusted to remain consistent with the benchmark protocols.

The overall evaluation was designed from a deployment-oriented perspective. In addition to transformation accuracy, this study explicitly considered registration robustness and practical feasibility under hardware limits. This setting is important because the proposed framework is intended for robotics perception and edge sensing on computation-limited platforms rather than only for high-performance desktop environments.

#### 4.1.2. Compared Methods

To provide a balanced comparison, the baseline methods were divided into two groups. The first group consisted of classical and efficient geometric registration methods, including representative correspondence-based and robust-estimation-based approaches, such as GC-RANSAC [51]. This group was used to evaluate whether the proposed training-free geometric pipeline provides practical gains over conventional lightweight methods. The second group consisted of recent learning-based registration methods that have shown strong benchmark performance, especially in low-overlap scenarios, including DGR [52] and PointDSC [53]. This grouping was adopted to establish a clearer academic dialogue between deployable geometric methods and high-performing learning-based methods.

For indoor benchmarks, the comparison focused on recent methods commonly reported on 3DMatch and 3DLoMatch, together with representative geometric baselines. For outdoor evaluation on KITTI, the comparison included robust geometric methods and representative learning-based methods whenever comparable results were available. Efficient geometric baselines were retained because they are directly relevant to the efficiency-oriented objective of the present study.

#### 4.1.3. Evaluation Metrics

To evaluate registration performance from multiple perspectives, this study reports translation error (TE), rotation error (RE), registration recall (RR), and runtime when appropriate. Translation error is defined as

(21) $$TE=\|\overset{ˆ}{t}-t^{*}{\|}_{2},$$

where $\overset{ˆ}{t}$ and $t^{*}$ denote the estimated and ground-truth translations, respectively. Rotation error is defined as

(22) $$RE=arccos\left(\frac{tr({\overset{ˆ}{R}}^{⊤}R^{*})-1}{2}\right)\cdot \frac{180}{\pi },$$

where $\overset{ˆ}{R}$ and $R^{*}$ denote the estimated and ground-truth rotations, respectively. Registration recall is computed as

(23) $$RR=\frac{1}{N_{test}}\sum_{n=1}^{N_{test}}\;\;1({TE}_{n}<\delta_{t}\wedge {RE}_{n}<\delta_{r}),$$

where $N_{test}$ is the number of test pairs, and $\delta_{t}$ and $\delta_{r}$ are the translation and rotation thresholds used by the corresponding evaluation protocol.

In addition to these metrics, point-wise residual measures such as RMSE may also be reported in related studies. However, RMSE alone can be relatively loose when used as the sole criterion in low-overlap and deployment-oriented registration, because small residuals do not necessarily imply correct transformation estimation, stable recall, or practical runtime behavior. For this reason, the present study emphasizes TE, RE, RR, runtime, and qualitative alignment results jointly rather than relying on a single error metric.

#### 4.1.4. Parameter Settings

The main parameter settings used in the proposed framework are summarized in Table 1. To maintain consistency with the geometric scale and sensing characteristics of different benchmarks, separate configurations were adopted for indoor and outdoor scenarios. In particular, 3DMatch and 3DLoMatch use a finer preprocessing resolution and smaller distance thresholds, whereas KITTI uses coarser downsampling and larger spatial thresholds to reflect large-scale outdoor LiDAR sensing conditions.

The indoor setting uses a voxel downsampling size of 5 cm and a feature extraction radius consistent with local geometric registration in cluttered indoor scenes. In contrast, the outdoor setting on KITTI uses a voxel size of 30 cm and correspondingly larger search radii for normal estimation, feature extraction, and robust correspondence verification. This distinction is necessary because the spatial scale, point density, and measurement range differ substantially between indoor RGB-D reconstructions and outdoor LiDAR scans.

For robust coarse estimation, the theoretical maximum RANSAC iteration budget was set to 2,000,000 for indoor datasets and 4,000,000 for KITTI. The larger iteration budget in the outdoor setting was adopted to accommodate the increased geometric scale and candidate ambiguity. Setting a large theoretical ceiling (e.g., 2 million) is a common practice across contemporary low-overlap registration pipelines to ensure the estimator is not artificially bottlenecked. We adopted this standard budget to maintain a fair comparison with existing methods, demonstrating that our performance gains fundamentally reflect the high quality of the candidate pairs preserved by ACE and purified by DGTG.

To ensure feasible execution on a resource-constrained 15 W edge CPU, this large budget acts strictly as a worst-case fallback cap. In practice, our framework implements a dual-stage scheduling strategy. An “elite sprint” stage is first performed exclusively on the top 30% elite candidates filtered by DGTG, with a strictly reduced cap (e.g., 100,000 iterations) and an early-stopping criterion. Because DGTG significantly increases the inlier ratio within this elite set, the estimation typically succeeds and terminates within only a few thousand iterations. Furthermore, because the underlying robust estimator relies on highly optimized backends, even an occasional fallback to the maximum budget does not induce catastrophic delays.

For fine refinement, the maximum number of UAMR iterations was set to 30 in indoor scenes, while an adaptive dual-strategy with 30 or 50 iterations was used on KITTI depending on the refinement radius and scene difficulty. The influence of key parameters is further analyzed in the parameter sensitivity and deployment analysis sections.

#### 4.1.5. Fairness and Deployment Considerations

For 3DMatch and 3DLoMatch, the compared baseline results were uniformly adopted from a single recent benchmark study when same-platform re-evaluation was unavailable. The proposed method was evaluated on a local GPU-free edge platform equipped with an Intel Core i5-8265U processor (15 W TDP), 8 GB RAM, and Windows 10, with GPU acceleration disabled. In contrast, the referenced indoor baseline results were reported in a substantially stronger desktop environment equipped with an Intel i7-14700F processor, 32 GB DDR5-5600 RAM, and an NVIDIA RTX 4060 Ti 8 GB GPU. Accordingly, the indoor comparison in this study focuses on benchmark-level registration accuracy under the reported evaluation protocols rather than on strict same-hardware runtime comparison.

For KITTI and the deployment-oriented analysis, the proposed method was evaluated on the same local GPU-free edge platform. The corresponding runtime observations are therefore reported to demonstrate practical deployment feasibility under limited hardware budgets. In this study, efficiency results are interpreted from a deployment perspective rather than as absolute cross-hardware runtime rankings.

This distinction is important because the central objective of the proposed framework is not only to achieve competitive registration performance, but also to remain deployable under strict hardware limits.

### 4.2. Benchmark Comparison on 3DMatch and 3DLoMatch

#### 4.2.1. Performance Evaluation on 3DLoMatch Benchmark

The quantitative results on 3DLoMatch are reported in Table 2. This benchmark is particularly important because it emphasizes low-overlap indoor registration, where true correspondences are sparse and mismatch-induced failure becomes more likely. From the perspective of deployment-oriented robotics perception, performance on 3DLoMatch is more indicative of practical robustness than standard-overlap evaluation alone.

As shown in Table 2, the proposed method achieves the highest registration recall on 3DLoMatch and also yields the lowest rotation and translation errors among the listed methods. This result is meaningful because 3DLoMatch directly stresses the low-overlap failure mode, in which early correspondence loss and false-match accumulation can severely affect rigid transformation estimation. The result suggests that the proposed ACE stage is effective in preserving potentially valid correspondences, while the DGTG stage improves candidate precision before coarse rigid estimation. For practical robotics perception, such low-overlap robustness is highly relevant because limited overlap is a common difficulty in real multi-view registration and mobile sensing.

Another important observation from Table 2 is that the proposed method remains competitive not only against classical baselines, but also against representative learning-based methods. This supports the central design objective of the present study. Rather than relying on training-dependent end-to-end refinement, the proposed framework improves correspondence coverage and geometric filtering quality within a lightweight geometric pipeline. The 3DLoMatch result therefore shows that training-free registration can remain effective under difficult indoor conditions when the method is explicitly structured for low-overlap robustness.

#### 4.2.2. Performance Evaluation on 3DMatch Benchmark

The quantitative results on 3DMatch are summarized in Table 3. Compared with 3DLoMatch, 3DMatch represents a less extreme indoor benchmark, and therefore serves as a useful complement for evaluating whether the proposed framework remains competitive under more standard registration conditions.

As shown in Table 3, the proposed method also performs strongly on 3DMatch. In particular, GeoRescue achieves the highest registration recall and the lowest rotation and translation errors among the listed methods. This indicates that the proposed training-free geometric framework is not restricted to difficult low-overlap cases, but also remains highly competitive under standard indoor benchmark settings. Taken together with the 3DLoMatch result, this suggests that the proposed method is able to maintain both robustness and accuracy across different overlap regimes.

From a methodological perspective, Table 3 also supports an important conclusion of this study: a carefully designed geometric pipeline can remain competitive even in a comparison that includes recent learning-based methods. This is consistent with the journal’s preference for methods that combine clear modular design with practical engineering significance. In the present case, the benchmark results indicate that candidate preservation, geometric topology gating, and uncertainty-aware refinement together provide an effective training-free alternative to more hardware-intensive solutions.

#### 4.2.3. Qualitative Comparison Under Low-Overlap Indoor Scenes

**Figure 8:**
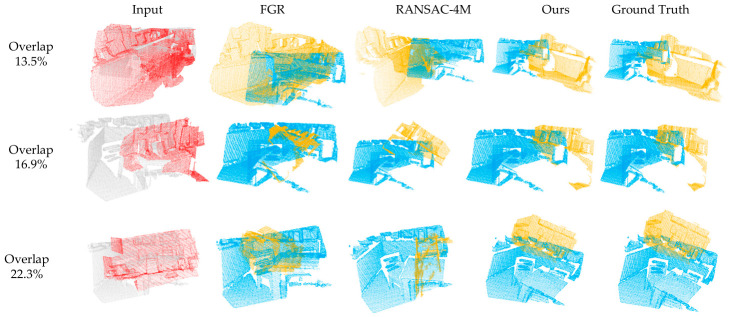
Qualitative comparison of registration results under low-overlap indoor scenes. The first column shows the input point clouds, where the red and gray colors denote the source and target point clouds, respectively. During registration, the target (gray) point cloud remains fixed while the source (red) point cloud is transformed. The subsequent columns present the registration results of different methods (FGR, RANSAC-4M, and the proposed method), followed by the ground-truth alignment. For visualization, the aligned source and target point clouds are shown in yellow and blue, respectively. The proposed method produces more accurate and globally consistent alignments, especially under challenging low-overlap conditions.

While Table 2 and Table 3 provide quantitative evidence, qualitative visualization is also important because registration behavior under low overlap is often easier to interpret in geometric space than through scalar metrics alone. Figure 8 presents representative indoor registration results under different overlap conditions, including conventional geometric baselines, the proposed method, and the corresponding ground-truth alignment.

Figure 8 shows that the proposed method produces more stable alignment under difficult overlap conditions than several conventional baselines. In moderate-overlap cases, multiple methods may recover visually plausible transformations, although the alignment quality still differs. As the overlap ratio decreases, however, conventional methods become more sensitive to ambiguous correspondences and local mismatch, whereas the proposed framework preserves better global consistency. This visual behavior is consistent with the design of the ACE and DGTG stages: the former enlarges the candidate pool to reduce early correspondence loss, and the latter suppresses geometrically inconsistent hypotheses before coarse rigid estimation.

The qualitative results also help clarify the practical meaning of registration recall. A method may partially align local regions while still fail to recover the correct global transformation. In contrast, the proposed method tends to maintain a more stable overall alignment across different overlap levels, which is consistent with its recall-oriented candidate expansion and geometry-aware filtering strategy.

#### 4.2.4. Performance Under Decreasing Overlap Ratios

**Figure 9:**
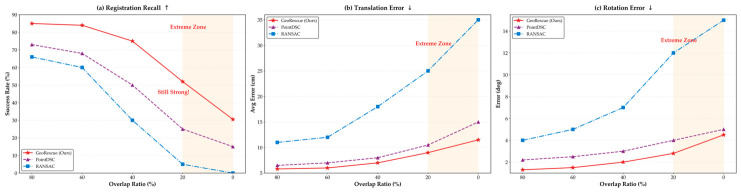
Registration performance under decreasing overlap ratios on 3DLoMatch.

To further analyze low-overlap robustness, the performance trend under progressively decreasing overlap ratios is shown in Figure 9. This experiment is particularly relevant to practical edge sensing scenarios because overlap degradation is a common source of registration failure in mobile perception and real-world data fusion.

Figure 9 reports the variation in registration recall, translation error, and rotation error as the overlap ratio decreases. As expected, all methods exhibit some level of degradation when overlap becomes smaller, because the number of valid correspondences decreases while the probability of mismatch increases. Nevertheless, the proposed method maintains a comparatively stable trend across a wider overlap range, which indicates that the proposed coarse-to-fine geometric pipeline remains effective under increasingly difficult matching conditions.

An important observation from Figure 9 is that low-overlap robustness cannot be adequately characterized by a single metric. The degradation pattern of recall is not always identical to that of translation or rotation error. This supports the evaluation strategy adopted in this study, in which TE, RE, RR, and qualitative alignment behavior are considered jointly. For deployment-oriented robotics perception, such joint evaluation is more informative than relying on only one residual-based indicator.

Overall, the results on 3DMatch and 3DLoMatch indicate that the proposed framework is not only effective under standard indoor benchmark conditions, but also remains robust when overlap becomes limited. This is especially relevant for deployment-oriented registration, where low overlap and correspondence ambiguity are common and where training-free geometric methods must remain reliable without access to heavy learned refinement.

### 4.3. Performance Evaluation on KITTI

#### 4.3.1. Quantitative Comparison on KITTI

To further evaluate the proposed framework in outdoor sensing scenarios, the method was tested on the KITTI odometry benchmark. Compared with indoor RGB-D reconstruction datasets, KITTI presents several additional challenges, including larger geometric scale, sparser LiDAR sampling, stronger viewpoint variation, and more severe sensitivity to frame-gap difficulty. From the perspective of robotics perception, this benchmark is particularly important because it better reflects deployment-oriented LiDAR registration in autonomous driving and outdoor mobile sensing environments.

As shown in Table 4, the proposed method achieves strong outdoor registration performance on KITTI while preserving its training-free geometric nature. In particular, the proposed framework yields the lowest translation error and rotation error among the listed methods, indicating that the UAMR refinement stage is effective in improving fine alignment quality after coarse geometric estimation. This result is important because outdoor LiDAR registration is typically affected by larger spatial scale, reduced overlap stability, and greater local structure ambiguity than indoor benchmark settings.

In terms of recall, the proposed method remains highly competitive, reaching 94.95%, which is close to the strongest robust geometric baseline in the table. Although SC-RANSAC attains a slightly higher recall, its time cost is substantially higher. From a deployment-oriented perspective, this comparison suggests that the proposed framework achieves a favorable balance between robustness, transformation accuracy, and practical computational cost. In other words, the method does not merely optimize one metric in isolation, but instead maintains strong overall performance under hardware-limited outdoor sensing conditions.

Another important observation from Table 4 is that local alignment alone is insufficient for large-displacement outdoor registration. ICP exhibits very low recall despite low residual error in successful cases, which indicates that local optimization without robust global initialization is unreliable in this setting. By contrast, the proposed framework maintains both high recall and low transformation error, which supports the effectiveness of the ACE–DGTG–UAMR pipeline for large-scale outdoor LiDAR registration.

#### 4.3.2. Performance Under Different Frame-Gap Difficulty Levels

**Figure 10:**
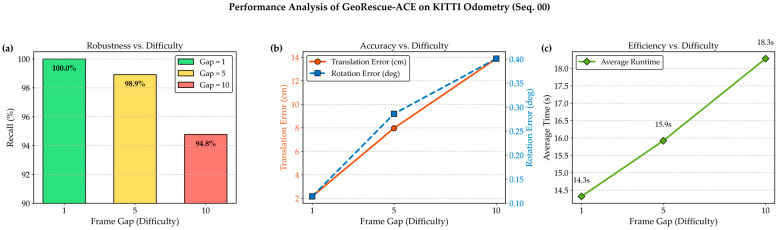
Performance analysis of GeoRescue-ACE on the KITTI Odometry dataset (Sequence 00) under varying frame-gap difficulty levels. (**a**) Robustness comparison showing the registration recall (%) across frame gaps of 1, 5, and 10, where the success rate decreases gradually from 100.0% to 94.8% as the viewpoint displacement increases; (**b**) Accuracy comparison illustrating the translation error (cm, red circles, left axis) and rotation error (deg, blue squares, right axis), both exhibiting a monotonic upward trend with larger frame gaps; (**c**) Efficiency comparison presenting the average runtime (s), which increases moderately from 14.3 s to 18.3 s, indicating predictable computational behavior under progressively more challenging conditions.

To further analyze outdoor robustness, the performance of the proposed method under different frame-gap difficulty levels is shown in Figure 10. In KITTI-based registration, increasing the frame gap typically enlarges viewpoint variation and reduces effective overlap, thereby making rigid registration more difficult. This setting is therefore useful for evaluating whether the proposed framework remains stable as outdoor registration conditions become more challenging.

Figure 10 reports the success rate, rotation error, translation error, and runtime of the proposed method under different frame-gap settings. As the frame gap increases, the registration task becomes more difficult because overlap decreases and geometric ambiguity increases. Nevertheless, the proposed method maintains relatively stable behavior across different difficulty levels. Although some degradation is still observed under larger frame gaps, the overall trend indicates that the proposed coarse-to-fine geometric pipeline remains effective in outdoor sensing scenarios with varying registration difficulty.

An important observation from Figure 10 is that the degradation in accuracy is not accompanied by uncontrolled runtime growth. This is relevant for practical robotics perception because outdoor deployment often requires not only robust registration, but also predictable computational behavior. In this sense, the proposed method shows favorable stability in both accuracy and runtime under progressively more difficult frame-gap settings.

Overall, the KITTI results indicate that the proposed framework can handle not only indoor benchmark registration, but also outdoor LiDAR alignment under larger spatial scale and increasing motion difficulty. This strengthens the deployment-oriented significance of the present study and supports the applicability of the proposed method to practical edge sensing tasks beyond standard indoor benchmarks.

### 4.4. Edge Deployment and Efficiency–Accuracy Trade-Off

A central objective of this study is not only to improve registration robustness, but also to maintain practical deployability under strict hardware limits. For this reason, the efficiency–accuracy trade-off of the proposed framework was further analyzed on a GPU-free 15 W edge platform. Compared with benchmark-only evaluation, this analysis is more directly related to practical robotics perception, where a method must remain usable under limited computational resources rather than only achieve strong accuracy in high-performance environments.

**Figure 11:**
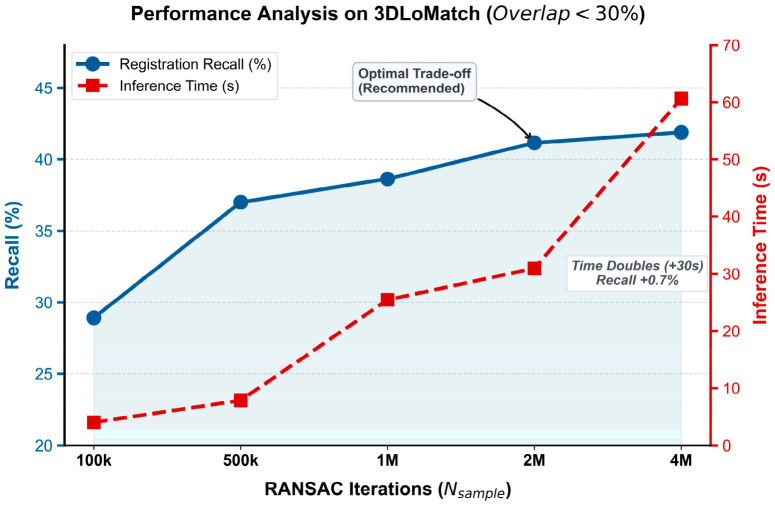
Efficiency–accuracy trade-off under different RANSAC iteration settings on 3DLoMatch.

Figure 11 presents the relationship between registration recall and inference time under different robust estimation settings on 3DLoMatch. This analysis is important because the iteration budget of the coarse estimation stage has a direct influence on both robustness and runtime. From a deployment perspective, the goal is not to maximize recall at any computational cost, but to identify an operating regime in which recall improvement remains meaningful relative to the additional runtime overhead.

Figure 11 shows that increasing the iteration budget generally improves registration recall, but the gain becomes progressively smaller as the number of iterations continues to grow. In contrast, the runtime cost increases more steadily. This indicates that there is a clear diminishing-return region in the efficiency–accuracy trade-off. For deployment-oriented registration, this observation is important because it shows that simply increasing the robust estimation budget is not an efficient strategy once the major geometric ambiguities have already been filtered by the ACE and DGTG stages.

Another important observation from Figure 11 is that the proposed framework preserves a useful operating range under limited hardware resources. Even on a GPU-free low-power platform, the method achieves competitive recall while maintaining practical runtime behavior. This result supports one of the main claims of the present study: training-free geometric registration can remain effective on resource-constrained hardware when candidate preservation, geometric filtering, and local refinement are jointly designed.

From a system-design perspective, the efficiency–accuracy behavior in Figure 11 also reflects the modular nature of the proposed framework. ACE improves candidate recall, DGTG reduces the burden of false-match propagation, and UAMR refines the final transformation without introducing a heavy learning-based inference stage. As a result, the method is able to maintain a favorable balance between robustness and computational cost under hardware limits.

These results further indicate that deployment-oriented registration should not be assessed only by the best achievable recall, but also by how efficiently that recall can be obtained on realistic hardware. In this sense, the proposed framework provides a practical operating point for edge LiDAR registration, which is more aligned with real-world perception constraints than purely accuracy-driven evaluation alone.

### 4.5. Ablation Study

To verify the specific contribution of each module, we systematically expanded the ablation study into two parts: an incremental contribution analysis (Table 5, ID 1–4) and a leave-one-out redundancy analysis (Table 5, ID 5–7). This approach allows us to isolate the impact of individual components while also assessing their collective synergy within the full GeoRescue framework.

The incremental analysis (ID 1–4) reveals the functional role of each module. Only ACE (ID 2) significantly increases the registration recall (RR) by 7.26% over the Vanilla Baseline (ID 1), confirming its effectiveness in candidate preservation. In contrast, Only DGTG (ID 3) exhibits a lower RR compared to the baseline but achieves the best efficiency (3.61 s), validating its role as a high-speed hypothesis filter. It is important to note that the efficacy of DGTG is inherently tied to the candidate pool quality; its lower recall when isolated confirms that DGTG is a precision-oriented filter that requires the candidate expansion provided by ACE to function optimally. Furthermore, Only UAMR (ID 4) validates that local manifold refinement is essential for final accuracy, yielding competitive TE and RE values even without the prior ACE/DGTG stages.

The redundancy analysis (ID 5–7) reinforces that each module is indispensable for the system’s robustness. Removing ACE (ID 5) makes the framework rely on raw reciprocal matches, which are fragile in low-overlap settings, leading to a noticeable performance drop. The absence of DGTG (ID 6) forces the RANSAC estimator to process a significantly higher ratio of false matches, thereby destabilizing the coarse estimate and increasing computation time. Most significantly, removing UAMR (ID 7) causes the most pronounced degradation in alignment precision, as the TE rises from 9.19 cm to 12.13 cm and the RE increases from 2.38° to 4.67°. This validates that our uncertainty-aware refinement is critical for converting coarse, potentially noisy estimates into stable, high-precision outcomes.

Finally, the Full GeoRescue (ID 8) achieves the most balanced performance across all metrics. The substantial performance leap between partial variants and the full framework confirms the synergistic integration of our multi-stage pipeline: ACE provides the necessary data volume, DGTG purifies this volume into a high-quality candidate subset, and UAMR performs the final precise alignment. Moreover, the runtime analysis demonstrates that our dual-stage scheduling ensures the full framework remains computationally feasible for real-time edge deployment, effectively bridging the gap between theoretical accuracy and practical edge-computing constraints.

### 4.6. Parameter Sensitivity Analysis

To further examine the stability of the proposed framework, the sensitivity of the ACE expansion factor K was analyzed on 3DLoMatch. This parameter is particularly important because it directly controls the size of the candidate correspondence pool generated in the ACE stage. If K is too small, potentially valid correspondences may be discarded too early, which weakens the robustness of subsequent geometric verification. If K is too large, the candidate pool becomes increasingly ambiguous and the computational burden of downstream filtering grows accordingly. Therefore, the selection of K reflects a practical trade-off between candidate coverage and computational efficiency.

**Figure 12:**
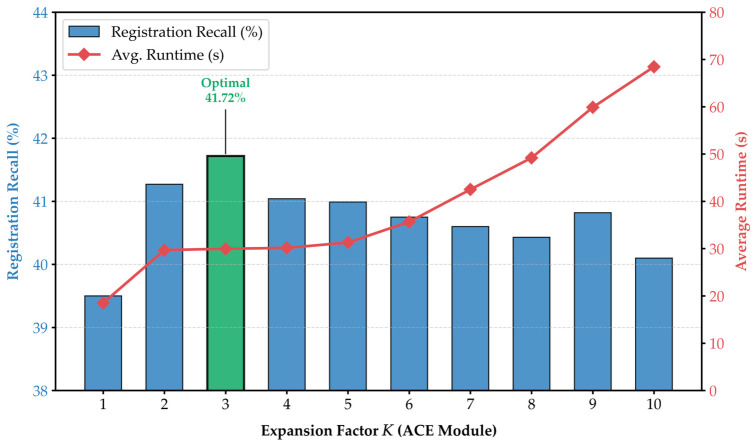
Sensitivity analysis of the ACE expansion factor K.

Figure 12 reports the variation in registration recall and average runtime as the expansion factor K changes. As shown in the figure, increasing K from a very small value initially improves registration recall because more potentially correct correspondences are preserved in the candidate set. This behavior is consistent with the design motivation of ACE: under low-overlap conditions, preserving a sufficiently rich correspondence pool is essential for maintaining robust downstream estimation.

However, the improvement does not continue indefinitely. After a certain range, the gain in recall becomes marginal, whereas the runtime increases more noticeably. This indicates that excessively enlarging the candidate pool introduces additional computational cost without producing a proportional increase in effective geometric support. In other words, a larger K does not necessarily imply better overall registration behavior once the major correspondence loss has already been alleviated.

The figure also shows that the proposed method exhibits a relatively stable operating region rather than extreme sensitivity to small parameter perturbations. This is important from a deployment perspective because methods intended for edge sensing should not depend on highly fragile parameter tuning. Instead, they should retain stable performance across a reasonable parameter interval while allowing practitioners to choose a value that matches the available hardware budget.

From the perspective of practical deployment, the optimal setting of K should be understood as a compromise between robustness and runtime rather than as a purely accuracy-driven choice. A moderate value provides sufficient candidate diversity for low-overlap registration while avoiding unnecessary downstream computation. This observation is consistent with the overall design philosophy of the proposed framework, which emphasizes efficiency-aware geometric robustness on computation-limited platforms.

Overall, the sensitivity analysis indicates that the ACE expansion factor is an important but manageable parameter. The proposed framework does not rely on extreme parameter settings to achieve its main gains. Instead, its performance advantage is maintained within a practically meaningful range of K, which supports the engineering usability of the method in real deployment scenarios.

### 4.7. Robustness Under Noise and Extreme Low-Overlap Conditions

To further evaluate the robustness of the proposed framework under adverse sensing conditions, additional experiments were conducted from two perspectives: sensitivity to increasing noise and qualitative behavior under extremely low-overlap scenarios. These analyses are important because practical robotics perception rarely operates under ideal geometric conditions. Instead, registration performance is often affected by sensor noise, local structural ambiguity, and severely reduced overlap between successive observations.

#### 4.7.1. Robustness Under Increasing Noise Levels

**Figure 13:**
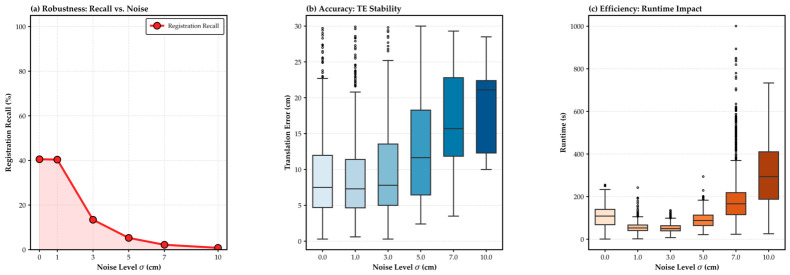
Registration robustness under increasing noise levels.

Figure 13 shows the behavior of the proposed method under different noise levels. This experiment is intended to evaluate whether the proposed coarse-to-fine geometric pipeline remains stable when point coordinates are progressively perturbed.

Figure 13 reports three aspects of performance under increasing noise: registration recall, translation error distribution, and runtime behavior. As expected, the registration task becomes more difficult as the noise level increases, because local geometric descriptors become less reliable and the correspondence space becomes more ambiguous. This tendency is reflected by the gradual decline in recall as the noise perturbation grows.

At the same time, the translation error distribution becomes wider under larger noise levels, indicating that local geometric uncertainty increasingly affects the final transformation estimate. This observation is consistent with the role of UAMR in the proposed framework. Although uncertainty-aware refinement improves local alignment quality, severe noise still reduces the reliability of neighborhood geometry and therefore limits the achievable refinement accuracy. In other words, refinement can mitigate noise effects to some extent, but cannot completely eliminate the structural degradation introduced at high noise levels.

The runtime trend in Figure 13 also provides an important deployment-oriented insight. Although runtime increases under more severe noise, the increase remains gradual rather than explosive. This suggests that the proposed framework degrades in a controlled manner under noisy sensing conditions. For practical edge deployment, such controlled degradation is important because it indicates that the method does not become computationally unstable even when the input quality deteriorates.

Taken together, the results in Figure 13 show that the proposed framework is sensitive to noise, as any geometric registration method would be, but still retains interpretable and stable behavior across a range of perturbation levels. This supports the practical value of the method for real sensing scenarios in which moderate geometric corruption is unavoidable.

#### 4.7.2. Qualitative Behavior Under Extreme Low-Overlap Conditions

**Figure 14:**
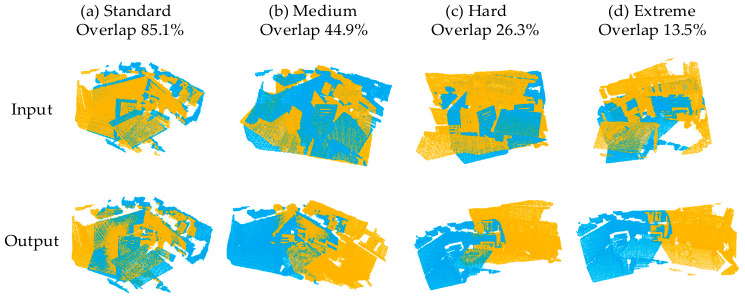
Qualitative registration results under different overlap difficulty levels. Yellow denotes the source point cloud (transformed by GeoRescue) and blue denotes the target point cloud (fixed).

To complement the quantitative robustness analysis, Figure 14 presents qualitative registration results under progressively more difficult overlap conditions, ranging from standard cases to extremely low-overlap scenes. This visualization is useful because extreme low-overlap failure is often easier to understand through geometric alignment patterns than through scalar indicators alone. It should be noted that such structural degradation under extreme sparsity is a common challenge, also observed in complex ULS-TLS forest canopy registration [54] and multi-modal environmental mapping [55].

Figure 14 shows that the proposed framework maintains reasonable alignment quality from standard overlap to more difficult hard-overlap cases, while the difficulty increases substantially in the extreme-overlap setting. As the available overlap decreases, the visible common structure between the source and target point clouds becomes progressively smaller, which directly weakens the amount of reliable geometric evidence available for rigid estimation. Under such conditions, even a robust correspondence expansion and filtering mechanism must operate with increasingly limited support.

Nevertheless, the qualitative results suggest that the proposed framework still preserves coherent transformation behavior across a broad difficulty range. This is consistent with the modular design of the method: ACE attempts to preserve candidate recall, DGTG suppresses grossly inconsistent hypotheses, and UAMR refines the final alignment when sufficient geometric support remains available. The visual results therefore provide intuitive evidence that the proposed framework degrades progressively rather than catastrophically when overlap becomes extremely limited.

From the perspective of deployment-oriented robotics perception, this behavior is important. In practical operation, the most useful method is often not the one that never fails, but the one that remains stable, interpretable, and predictable as conditions worsen. The results in Figure 14 suggest that the proposed framework exhibits this kind of progressive degradation pattern, which is desirable for real-world low-power sensing systems operating in challenging geometric environments.

Overall, the robustness analysis under noise and extreme low-overlap conditions indicates that the proposed framework maintains stable geometric behavior beyond standard benchmark settings. This strengthens the practical significance of the present study and supports the applicability of the method to real edge sensing scenarios in which uncertainty and overlap degradation are common.

### 4.8. Statistical and Complexity Analysis

To further examine the practical behavior of the proposed framework beyond average benchmark metrics, a statistical analysis was conducted from multiple perspectives, including robustness, runtime distribution, gated feature count, error cumulative behavior, and the correlation between intermediate geometric filtering and computational cost. This analysis is important because deployment-oriented registration should not be judged only by mean recall or average transformation error. In practical robotics perception, it is equally important to understand how stable the method is across samples, how runtime is distributed, and how internal geometric filtering behavior relates to final computational burden.

**Figure 15:**
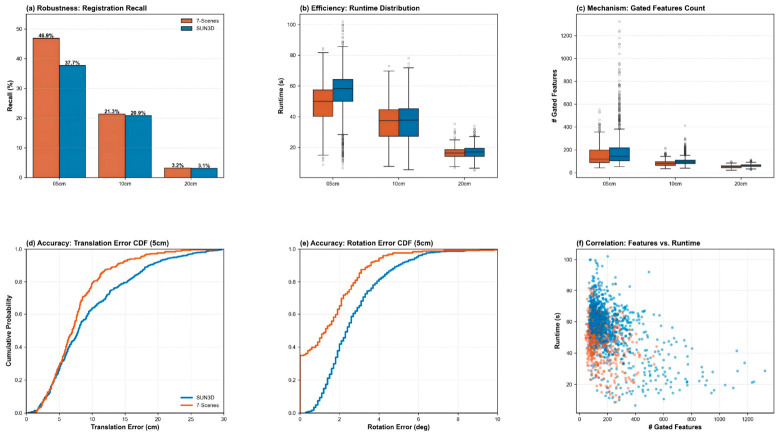
Statistical analysis of robustness, runtime distribution, gated feature count, and error behavior.

Figure 15 provides a more detailed view of the statistical behavior of the proposed framework on representative indoor data. Figure 15a–c summarize robustness, runtime, and gated feature statistics under different thresholds, while Figure 15d–f show the cumulative and correlational behavior of translation error, rotation error, and runtime. Taken together, these results complement the benchmark-level averages reported earlier and provide a more engineering-oriented understanding of how the method behaves in practice.

The robustness plot in Figure 15a shows that registration recall decreases as the acceptance threshold becomes stricter. This is expected because fewer correspondences survive the geometric gating process when the threshold is tightened. At the same time, the figure indicates that the proposed framework still preserves useful recall under moderate threshold settings, which suggests that the gating mechanism is not merely discarding candidates aggressively, but is filtering them in a controlled manner.

Figure 15b presents the runtime distribution under different settings. Instead of focusing only on average runtime, this panel reveals the spread, skewness, and outlier behavior of the computational cost. This is important for deployment-oriented systems because average time alone does not capture whether a method occasionally produces prohibitively slow cases. The observed runtime distributions indicate that the proposed method remains statistically manageable across settings, although more difficult or less restrictive configurations naturally produce a wider time spread.

Figure 15c reports the number of gated features retained after geometric filtering. This quantity is closely related to the intermediate complexity of the registration pipeline. When more features survive the DGTG stage, the subsequent estimation and refinement process may gain robustness, but it also tends to increase computational burden. Therefore, this panel provides useful evidence that the proposed method operates through a measurable internal trade-off rather than through a black-box inference mechanism.

The cumulative distribution plots in Figure 15d,e further clarify the error behavior of the proposed framework. Compared with isolated average values, cumulative curves show how often the method stays below a given translation or rotation error threshold. This is more informative for practical deployment because it reveals not only the center of the error distribution, but also the proportion of easy, moderate, and difficult cases. The observed CDF behavior indicates that the proposed framework maintains relatively concentrated error distributions in a substantial portion of the evaluated samples, which supports its practical stability.

Finally, Figure 15f shows the correlation between the number of gated features and runtime. This panel provides a direct statistical interpretation of the internal computational mechanism of the method. In general, larger gated feature counts tend to be associated with higher runtime, which is consistent with the design of the geometric pipeline: stronger candidate preservation and broader hypothesis support can improve robustness, but they also increase computational load. This relationship is important because it quantitatively supports the engineering trade-off discussed throughout this study.

From the perspective of computational complexity, the proposed framework remains interpretable because its main cost arises from explicit geometric operations rather than hidden learned inference. ACE incurs the cost of bidirectional candidate construction, DGTG introduces pairwise geometric consistency evaluation and hypothesis gating, and UAMR adds local covariance-guided iterative refinement. Although these stages introduce additional computation compared with very simple baselines, they also provide clear functional gains in correspondence preservation, hypothesis purification, and final alignment accuracy. This trade-off is particularly meaningful for edge deployment, where a method must justify its computational cost through improved robustness and accuracy.

Overall, the statistical and complexity analysis in Figure 15 supports the view that the proposed framework is not only accurate on average, but also behaviorally stable, computationally interpretable, and practically controllable. These properties are important for deployment-oriented LiDAR registration, where robustness, predictability, and engineering transparency are often as important as headline benchmark performance.

## 5. Discussion

The results presented in Section 4 support three main observations. First, the proposed framework remains competitive on both standard-overlap and low-overlap indoor benchmarks. Second, it generalizes to outdoor LiDAR registration on KITTI without relying on model training. Third, it preserves practical deployment value under GPU-free edge hardware constraints. Taken together, these results suggest that the proposed method is not merely a benchmark-oriented registration pipeline, but a deployment-oriented geometric framework for robotics perception under hardware limits.

A central implication of this study is that training-free registration remains a viable direction when the method is carefully designed around candidate preservation, geometric filtering, and uncertainty-aware refinement. In recent years, point cloud registration research has increasingly emphasized learning-based correspondence refinement and feature matching. These approaches can achieve strong benchmark performance, especially under complex appearance and overlap conditions. However, they also tend to increase dependency on training data, model availability, GPU support, and inference overhead. The present results indicate that a purely geometric pipeline can still remain highly competitive when its internal stages are explicitly structured to address the main failure points of low-overlap registration.

In particular, the role separation among ACE, DGTG, and UAMR appears to be important. ACE improves correspondence recall at the early stage, which is essential when overlap is limited and one-way matching is too fragile. DGTG then suppresses false or weakly supported hypotheses through lightweight geometric consistency checks, thereby reducing the burden on coarse rigid estimation. Finally, UAMR refines the solution by explicitly accounting for local anisotropic uncertainty during optimization. This modular structure is consistent with the engineering preference of the target journal: each module addresses a clearly defined subproblem, and the overall gain arises from the interaction of these modules rather than from a single opaque component.

Another important point is the interpretation of evaluation metrics. In this study, TE, RE, RR, runtime, and qualitative alignment behavior were emphasized jointly. This choice is intentional. In practical low-overlap registration, no single metric can fully describe method quality. For example, RMSE or point-wise residual measures may appear favorable even when the recovered transformation is not sufficiently robust or when the method fails frequently under difficult overlap conditions. Similarly, high recall alone does not guarantee low transformation error, and low average error alone does not reflect runtime feasibility. Therefore, the present study adopts a more application-oriented evaluation strategy, which is better aligned with deployment scenarios in robotics perception.

The deployment significance of the proposed framework should also be interpreted carefully. For indoor benchmarks, the comparison mainly reflects benchmark-level registration accuracy, because same-platform re-evaluation was not available for all referenced baselines. For KITTI and the additional deployment analysis, the proposed method was directly evaluated on a GPU-free 15 W edge platform. Therefore, the efficiency-related conclusions of this study should be understood primarily as hardware-aware deployment observations rather than as unconditional cross-hardware speed rankings. This distinction is important for maintaining a fair interpretation of practical performance.

From an application perspective, the proposed framework is particularly relevant to scenarios in which compute, memory, and power budgets are constrained. Examples include mobile robots, lightweight autonomous platforms, low-power mapping systems, and embedded sensing nodes that require geometric registration without reliable access to high-end GPUs. In such settings, the practical question is not only whether a method can achieve the best benchmark score, but whether it can provide sufficiently robust registration with predictable and manageable computational cost. The present results suggest that the proposed framework addresses this requirement reasonably well.

At the same time, several limitations should be acknowledged. First, the method still degrades when overlap becomes extremely small or when geometric noise becomes severe. This behavior is expected, because the available structural evidence for rigid registration becomes increasingly limited in these cases. Second, although the proposed framework is more deployment-oriented than many training-dependent alternatives, its runtime can still increase noticeably when the candidate pool becomes large or when robust estimation requires a high iteration budget. Third, the current framework is still built around handcrafted geometric processing and does not exploit potentially useful prior information from semantics, temporal continuity, or task-specific scene regularities.

These limitations point to several directions for future work. One possible extension is to introduce adaptive scheduling mechanisms that more directly control candidate expansion, geometric gating, and refinement depth according to scene difficulty. Furthermore, exploring global geo-positional awareness [56] or robust ground segmentation priors [57] could enhance the method’s adaptability in large-scale autonomous driving environments. Another direction is to combine the present training-free backbone with lightweight learned priors in a controlled hybrid manner, provided that the deployment cost remains acceptable for edge platforms. It may also be valuable to explore stronger hardware-aware optimization strategies, such as approximate correspondence pruning, more efficient graph filtering, or parallelized local refinement, in order to further improve real-time usability on embedded platforms.

Overall, the proposed study contributes to the current point cloud registration literature by showing that a pure geometric, training-free framework can still provide strong and practically meaningful performance when it is explicitly designed for low-overlap robustness and edge deployment. Rather than competing only in terms of isolated benchmark scores, the method aims to provide a balanced solution for robotics perception under realistic hardware constraints, which is the main practical message of this work.

## 6. Conclusions

This study presented GeoRescue, a training-free geometric LiDAR point cloud registration framework designed for resource-constrained edge platforms. The proposed method addresses low-overlap registration through a coarse-to-fine pipeline composed of three modules: Asymmetric Correspondence Expansion (ACE), Dynamic Geometric Topology Gating (DGTG), and Uncertainty-Aware Manifold Refinement (UAMR). In contrast to training-dependent registration pipelines, the proposed framework emphasizes explicit geometric reasoning, lightweight computation, and deployment feasibility under hardware limits.

Experimental results on 3DMatch, 3DLoMatch, and KITTI showed that the proposed method achieves competitive performance across both indoor and outdoor registration scenarios. In particular, the framework demonstrated strong robustness under low-overlap conditions, favorable transformation accuracy, and practical deployment value on a GPU-free 15 W edge platform. The ablation and sensitivity analyses further showed that ACE, DGTG, and UAMR play complementary roles in improving candidate preservation, geometric filtering, and fine alignment refinement, respectively. Additional robustness, trade-off, and statistical analyses also supported the practical stability and engineering interpretability of the proposed framework.

Overall, the results suggest that training-free geometric registration remains a viable and meaningful direction for robotics perception when the method is explicitly designed around low-overlap robustness and limited computational resources. The main contribution of this work is therefore not only a competitive registration pipeline, but also a deployment-oriented design perspective for edge LiDAR registration. Future work will focus on more adaptive scheduling strategies, stronger hardware-aware optimization, and lightweight hybrid extensions that preserve the practical advantages of the present geometric framework.

## Figures and Tables

**Table 1:** Main parameter settings used in the proposed framework for indoor and outdoor scenarios.

Parameter Category	Indoor Scenarios (3DMatch/3DLoMatch)	Outdoor Scenario (KITTI Odometry)
Voxel downsampling size	5 cm	30 cm
Normal estimation search radius	0.1 m	0.6 m
Maximum nearest neighbors for normal estimation	30	30
FPFH feature search radius	0.25 m	1.5 m
Maximum nearest neighbors for FPFH feature extraction	100	100
Maximum RANSAC iterations	2,000,000	4,000,000
RANSAC correspondence distance threshold	7.5 cm	45 cm
UAMR refinement search radius	10 cm	1.2 m/5.0 m (adaptive)
Maximum UAMR refinement iterations	30	30/50 (adaptive dual-strategy)

**Table 2:** Quantitative comparison on 3DLoMatch.

3DLoMatch	Category	Hardware	RR (%)	RE (°)	TE (cm)
RANSAC-1M [6]	Traditional	CPU	0.67	10.27	15.06
RANSAC-4M [6]	Traditional	CPU	0.45	10.39	20.03
MAC [46]	Traditional	CPU	40.88	3.66	9.45
FastMAC@50 [47]	Traditional	GPU	38.46	4.04	10.47
DGR [52]	Deep Learning	GPU	19.88	5.07	13.53
PointDSC [53]	Deep Learning	GPU	20.38	4.04	10.25
Ours	Traditional	CPU	41.27	2.30	9.06

**Table 3:** Quantitative comparison on 3DMatch.

3DMatch	Category	Hardware	RR (%)	RE (°)	TE (cm)
RANSAC-1M [6]	Traditional	CPU	64.20	4.05	11.35
RANSAC-4M [6]	Traditional	CPU	66.10	3.95	11.03
GC-RANSAC [51]	Traditional	CPU	67.65	2.33	6.87
TEASER++ [8]	Traditional	CPU	75.48	2.48	7.31
MAC [46]	Traditional	GPU	83.90	2.11	6.80
FastMAC@50 [47]	Traditional	GPU	82.87	2.15	6.73
3DRegNet [28]	DeepLearning	GPU	26.31	3.75	9.60
DGR [52]	DeepLearning	GPU	32.84	2.45	7.53
PointDSC [53]	DeepLearning	GPU	72.95	2.18	6.45
Ours	Traditional	CPU	84.84	1.29	5.88

**Table 4:** Quantitative comparison on the KITTI odometry benchmark for outdoor LiDAR registration.

Method	Type	Recall (%)	TE (cm)	RE (°)	Time (s)
ICP [5]	Geometric	0.90	8.30	0.1266	1.868
FGR [7]	Geometric	27.93	34.66	0.6594	15.909
RANSAC [6]	Geometric	94.84	18.23	0.6445	24.918
GC-RANSAC [51]	Geometric	96.94	19.11	0.6449	35.667
Ours	Geometric	94.95	13.89	0.4001	21.738

**Table 5:** Quantitative ablation and modular contribution analysis of GeoRescue on the 3DLoMatch dataset. The checkmark (✔) denotes that the corresponding module (ACE, DGTG, or UAMR) is included in the evaluated variant.

ID	Variant	ACE	DGTG	UAMR	RR (%)	TE (cm)	RE (°)	Time (s)
1	Vanilla Baseline				32.40	11.85	3.42	18.40
2	Only ACE	✔			39.66	10.54	2.91	20.45
3	Only DGTG		✔		16.76	12.10	4.05	3.61
4	Only UAMR			✔	40.22	9.68	2.55	17.97
5	w/o ACE		✔	✔	40.26	9.26	2.42	9.88
6	w/o DGTG	✔		✔	41.00	9.11	2.42	15.66
7	w/o UAMR	✔	✔		36.83	12.13	4.67	16.66
8	GeoRescue (Full)	✔	✔	✔	42.50	9.19	2.38	16.80

## Data Availability

The 3DMatch and 3DLoMatch datasets used in this study are publicly available from the 3DMatch project website (http://3dmatch.cs.princeton.edu/ (accessed on 25 May 2026)). The KITTI Odometry dataset is publicly available from the KITTI Vision Benchmark Suite (http://www.cvlibs.net/datasets/kitti/eval_odometry.php (accessed on 25 May 2026)). The source code, test scripts, and benchmark configuration files used in this study are publicly available at the GeoRescue repository: https://github.com/ZK131/GeoRescue (accessed on 25 May 2026).

## Abbreviations

The following abbreviations are used in this manuscript:

3DRegNet	3D Registration Network
ACE	Asymmetric Correspondence Expansion
CDF	Cumulative Distribution Function
CPU	Central Processing Unit
DGR	Deep Global Registration
DGTG	Dynamic Geometric Topology Gating
FastMAC	Fast Maximal Clique
FGR	Fast Global Registration
FPFH	Fast Point Feature Histograms
GC-RANSAC	Graph-Consensus Random Sample Consensus
GPU	Graphics Processing Unit
ICP	Iterative Closest Point
IQR	Interquartile Range
KNN	K-Nearest Neighbors
LiDAR	Light Detection and Ranging
PointDSC	Point Deep Spatial Consistency
RANSAC	Random Sample Consensus
RE	Rotation Error
RGB-D	Red Green Blue-Depth
RR	Registration Recall
SC-RANSAC	Spatial Consistency Random Sample Consensus
SLAM	Simultaneous Localization and Mapping
SVD	Singular Value Decomposition
TE	Translation Error
TDP	Thermal Design Power
TEASER++	Tensorial Estimation of Absolute Pose for Efficient Registration++
UAMR	Uncertainty-Aware Manifold Refinement
